# Severe metabolic accumulation of VV116 in kidney transplant patients with impaired renal function: a case series report

**DOI:** 10.3389/fimmu.2024.1501813

**Published:** 2025-01-17

**Authors:** Jiaying Zhang, Yuan Gao, Xiaoyun Miao, Wei Wang, Zhongkai Zhou, Yunyi Gao, Liwei Liu, Menghua Wu, Ke Ma, Ling Zhou, Yan Yang, Sha Meng, Yingmei Feng, Zhuorui Zhao, Wei Liu, Danlei Mou, Zixin Kang, Lianchun Liang, Zhongjie Hu

**Affiliations:** ^1^ Department of Infectious Disease, Beijing You’An Hospital, Capital Medical University, Beijing, China; ^2^ Fourth Department of Liver Disease, Beijing You’An Hospital, Capital Medical University, Beijing, China; ^3^ Department of Critical Care Medicine, Cangzhou Central Hospital, Cangzhou, Hebei, China; ^4^ Department of Radiology, Beijing You’An Hospital, Capital Medical University, Beijing, China; ^5^ Safe Transfusion Lab, Beijing Red Cross Blood Center, Beijing, China; ^6^ Department of Urology, Beijing Hospital of Traditional Chinese Medicine, Capital Medical University, Beijing, China; ^7^ Department of Bioanalysis, United-Power Pharma Tech Co., Ltd, Shanghai, China; ^8^ Department of Science and Technology, Beijing You’An Hospital, Capital Medical University, Beijing, China; ^9^ Department of Medicine, Xenorm MedInfo Center, Beijing, China; ^10^ Department of Pharmacy, Beijing You’An Hospital, Capital Medical University, Beijing, China; ^11^ Liver Disease Center, Beijing You’An Hospital, Capital Medical University, Beijing, China

**Keywords:** COVID-19, organ transplant, kidney dysfunction, drug accumulation, VV116

## Abstract

The treatment of COVID-19 in the post-transplant individuals is challenging, primarily due to the drug-drug interaction between nirmatrelvir/ritonavir and tacrolimus. Deuremidevir hydrobromide tablets (VV116) is an orally small molecule agents target SARS-CoV-2 RdRp and inhibits viral replication. It may have a low likelihood of drug-drug interactions and has a potential to provide new treatment option. We described three cases of renal transplant patients with concomitant impaired renal function who developed COVID-19 pneumonia and were treated with VV116. Despite varying degrees of drug accumulation, these patients achieved rapid viral clearance and showed prompt improvement in pneumonia symptoms. Notably, tacrolimus blood concentrations remained within the therapeutic range throughout treatment, and no clinically significant adverse events were observed despite the drug accumulation.

## 1 Introduction

Post solid-organ transplant patients pose a challenge in the treatment of COVID-19. Nirmatrelvir/ritonavir (Paxlovid) has been established as a highly effective oral antiviral for COVID-19 treatment. However, its use in patients receiving immunosuppressants, such as tacrolimus, is limited by significant drug-drug interactions arising from the ritonavir component of the combination (1). VV116 is a COVID-19 antiviral drug targeting the RdRp, its innovative design bestows it with a potent and stable capabilities to inhibit the replication of SARS-CoV-2 as well as low drug-drug interaction (2). Herein, we share a case series of COVID-19 pneumonia patients treated with VV116 and review the relevant literature. All patients provided written informed consent before receiving VV116.

## 2 Case description

### 2.1 Case 1

**Figure 1:**
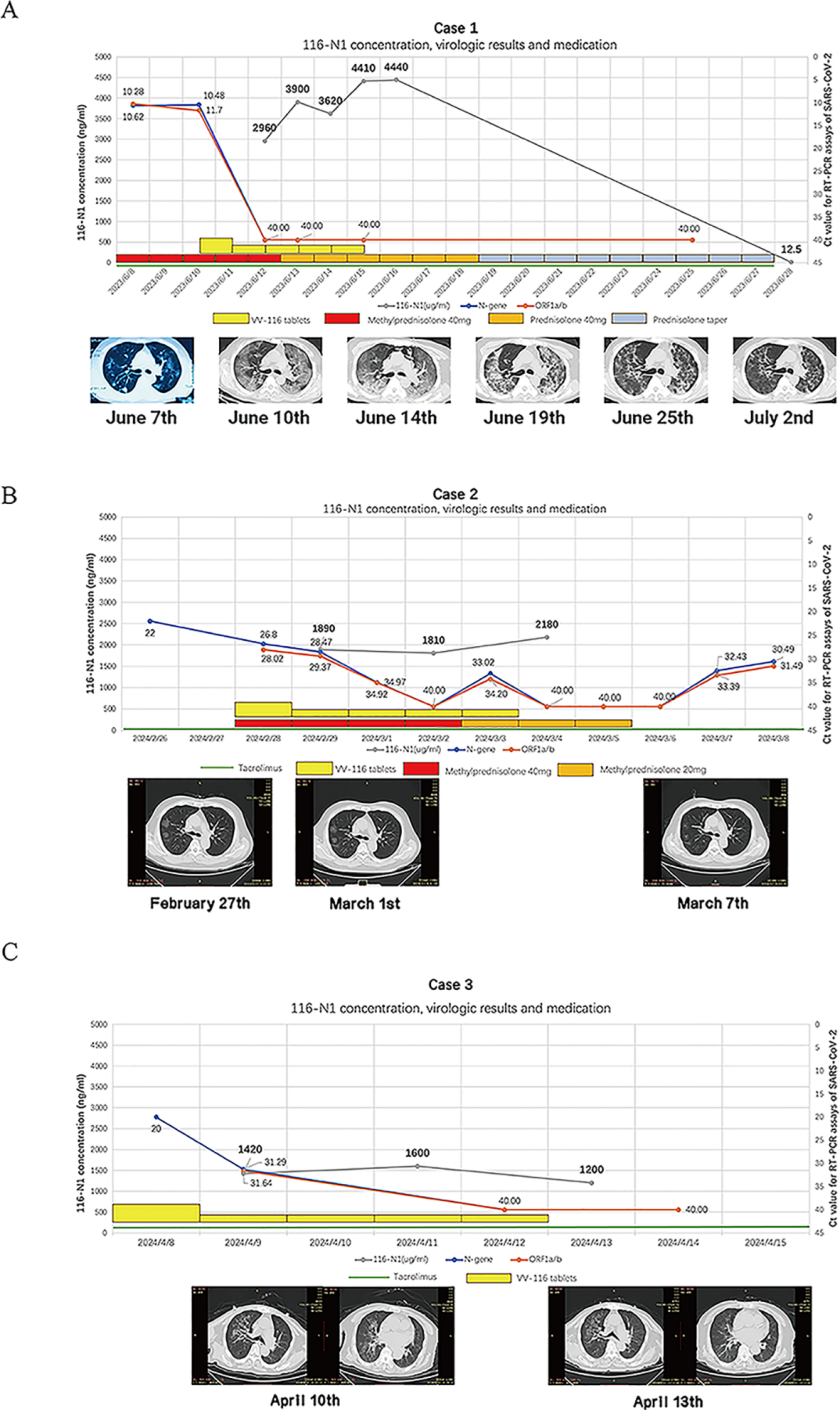
**(A)**. VV116-N1 concentration, virologic results and medicine administration for case 1, Serial chest imaging by computed tomography (CT), the image on June 7th was from another hospital and taken with a mobile phone camera. **(B)**. VV116-N1 concentration, virologic results and medicine administration for case 2, the Ct. value of N-gene on February 26th was from another hospital, Serial chest imaging by computed tomography (CT), the image was taken on February 27th, March 1st and March 7th. **(C)**. VV116-N1 concentration, virologic results and medicine administration for case 3, the Ct. value of N-gene on April 8th was from another hospital, Serial chest imaging by computed tomography (CT), the image was taken on April 10 th and April 13 th.

A 59-year-old male patient, underwent renal transplantation surgery 20 years ago and was receiving anti-rejection medications including tacrolimus, azathioprine, and prednisone. On June 1, 2023, the patient developed a fever with a peak temperature of 39°C, along with chills and a cough. On June 4 2023, a rapid antigen test for COVID-19 returned a positive result. On June 7, the patient experienced chest tightness. On June 8, the patient was admitted to the hospital. As the patient refused to stop taking immunosuppressants, especially tacrolimus, doctors were hesitant to use the available antiviral drug – nirmatrelvir/ritonavir for treatment. On June 10, the patient presented with severe respiratory distress and had an oxygen saturation measured by pulse oximetry of 77% while using an oxygen reservoir mask. The chest CT-scan revealed the progression of numerous bilateral ground-glass opacities with consolidation in localized regions (**Figure 1A**). Meanwhile, creatinine level elevated to 298 umol/L and required non-invasive ventilation support. On June 11, VV116 was administered, with an initial dose of 600 mg twice daily (day 1), followed by 300 mg twice daily for four days (day 2 to day 5) (**Figure 1A**). After the patient received two doses of VV116, a nasal swab was collected and yielded negative result (Ct value over 40). Blood samples were collected one hour before the second, fourth, sixth, and eighth doses for trough concentration monitoring and monitored drug concentration of VV116-N1, the metabolite of VV-116. The concentration of VV116-N1 were reported with drug accumulation (**Figure 1A**). His medication, Ct value, pulmonary CT and drug concentration of VV116-N1 were shown (**Figure 1A**). After 5 days of treatment with tacrolimus, plasma concentration test for tacrolimus showed a result of 5.83 ng/ml. 12 days after the last dose, plasma concentration of VV116 showed trace amounts of residual metabolites (116-N1 concentration was 12.5 ng/ml). Based on the difference of 116-N1 concentration and measurement interval, the drug half-life was calculated to be about 2.05 days (49.2 hours). No significant hematological abnormalities were noted during hospitalization, but a transient elevation in transaminase levels. (**Supplementary Figure S1**).

### 2.2 Case 2

A 50-year-old male was diagnosed with IgA nephropathy in 2012 and underwent kidney transplantation in January 2023. Post-transplant, he was maintained on tacrolimus and methylprednisolone. In February 2024, the patient developed a fever with a peak temperature of 39°C and tested positive for SARS-CoV-2 RT-PCR. On admission (day 4 of illness), his oxygen saturation was 92% when breathing ambient air, and Chest CT imaging revealed typical COVID-19 pneumonia (**Figure 1B**). The patient had a serum creatinine level of 152 µmol/L, and a tacrolimus trough level of 3.4 ng/ml. The patient refused to discontinue tacrolimus. VV116 was administered at half the standard dose (300 mg BID on day 1, followed by 200 mg in the morning and 100 mg in the evening for the next four days). This dose reduction was decided upon after clinical discussions based on observations of drug accumulation in case 1. His medication, Ct value, pulmonary CT and drug concentration of VV116-N1 were shown (**Figure 1B**). After the 10th dose of VV116, VV116 trough concentration monitoring indicated drug accumulation, but tacrolimus levels remained stable (4.4 ng/ml), and there were no significant fluctuations in liver or kidney function. During hospitalization, there were no significant fluctuations in liver and kidney function or blood routine test results. At discharge, the serum creatinine level was 106 µmol/L.

### 2.3 Case 3

An 84-year-old male patient, 20 years post-kidney transplantation, had experienced elevated creatinine levels for the past 6 years, with levels exceeding 130 µmol/L in the past year. At the time of admission (April 2024), he was on tacrolimus. The patient was admitted on the second day of illness onset, with a peak temperature of 38°C, oxygen saturation of 94% on room air, and a SARS-CoV-2 Ct value of 20.0 (N-gene). Chest CT revealed a typical viral pneumonia appearance due to COVID-19 (**Figure 1C**). The patient had a serum creatinine (Cr) level of 205 µmol/L and a tacrolimus trough level of 5.8 ng/ml. On the day of admission, VV116 was administered at half the standard dose, this dose reduction was decided upon after clinical discussions based on observations of drug accumulation in case 1. After five days of VV116 treatment, the patient RT-PCT test turned negative. We monitored the trough concentrations of VV116 after the 1st, 5th, and 9th doses, which were 1420 ng/ml, 1600 ng/ml, and 1200 ng/ml, respectively (**Figure 1C**). On day 7 of admission, the tacrolimus trough concentration was 5.3 ng/ml. At discharge, the patient’s serum creatinine level decline to 140 µmol/L. Blood tests showed no significant abnormalities in liver function.

## 3 Discussion

We report this case series with SARS-CoV-2 Omicron variant infection in kidney transplant patients with severe renal impairment. The infection was characterized by sustained viral replication and progression to severe pneumonia. The emergence of hypoxemia in the early stages of hospitalization may indicate a trajectory toward adverse outcomes (3). Solely utilizing corticosteroids without concurrent viral control probably be inadequate in halting the progression of the disease (4). In these kidney transplant cases with kidney function impairment, exploratory dose adjustments were implemented based on drug accumulation and therapeutic drug monitoring thresholds, VV116 can effectively inhibit SARS-CoV-2 in this specific population. VV116, a deuterated derivative of remdesivir, is designed for oral administration and demonstrates effective bioavailability, addressing the limitations of injectable remdesivir. VV116 inhibits the replication of SARS-CoV-2 by targeting the viral RNA-dependent RNA polymerase, similar to remdesivir. It competes with NTP substrates, effectively halting viral RNA elongation (5).Although patients taking immunosuppressants after solid organ transplantation may take several weeks to negative conversion (6, 7), these patients achieved a rapid virological response after receiving VV116 treatment, followed by full recovery. To our knowledge, this is the first case series reporting the use of VV116 in a solid organ transplant recipient with renal dysfunction.

Complete clearance of SARS-CoV-2 is crucial for improving clinical outcomes in such immunosuppressed patients (8), particularly as these individuals may experience delayed viral clearance (9, 10). The Mpro targeted by Paxlovid has been widely recognized as an ideal therapeutic target and has demonstrated potent inhibitory efficacy against SARS-CoV-2 in numerous studies (11). However, for organ transplant patients, tacrolimus needs to be discontinued at least 12 hours before using Paxlovid. However, this may not ensure that the concentration of tacrolimus will not increase significantly (12), and it could lead to adverse effects if tacrolimus is not discontinued timely (13). Hence, alternative COVID-19 antiviral treatments are necessary for organ transplant patients. In this case, the patient was treated with tacrolimus while receiving VV116. Although VV116-N1 concentration increased significantly, the trough concentration of tacrolimus remained within the normal range on the fifth day. This case suggests that VV116 has the potential to be an option for organ transplant patients who are currently receiving tacrolimus, without requiring a change of immunosuppressants doses.

Our literature review demonstrate that several case reports and observational studies have documented the risk of tacrolimus toxicity when used concomitantly with nirmatrelvir/ritonavir, remdesivir, chloroquine and lopinavir/ritonavir (Tables 1 and 2) among organ transplantation individuals. Remdesivir has been shown to inhibit certain cytochrome P450 enzymes, including CYP3A4, which can reduce tacrolimus clearance and thus elevate its plasma concentration, increasing the risk of adverse effects like nephrotoxicity and (14). Ritonavir, a component of Paxlovid, is a potent CYP3A4 inhibitor and can significantly affect the metabolism of tacrolimus, increasing tacrolimus concentrations in the body. This could increase the risk of tacrolimus toxicity, including nephrotoxicity and hepatotoxicity (15).Hence, it is necessary to find an alternative antiviral agent for these patients. In our case series, the patient was treated with tacrolimus while receiving VV116. Although VV116-N1 concentration increased significantly, the trough concentration of tacrolimus remained within the normal range. This means that VV116 does not affect the blood concentration of tacrolimus, therefore not requiring a change of concurrently used immunosuppressants.

VV116 was principally excreted through kidney in the form of metabolite VV116-N1, renal impairment can significantly affect its metabolic rate. As presented in our case series, after receiving a standard course of VV116, the trough concentration of was over 10 times higher than in the general population (the trough concentrations of 116-N1 following multiple administration of 200 mg Q12H, 400 mg Q12H, 600mg Q12H were 242 - 345 ng/mL, 559 - 766  ng/mL, and 721 - 1011  ng/mL, respectively), while the half-life of metabolite was found to be approximately 8 times longer than general population (4.80 - 6.95 hour) in Case 1 (16). When VV116 was administered a half-dose regimen, the blood drug concentration in patients with renal impairment fluctuated between 1200-2180 ng/mL, which remained significantly above the EC_90_ level (186.5 ng/mL). For the transient elevation of transaminases in Case 1, in a large head-to-head study, the incidence of hepatic function abnormalities was 5.7% in the VV116 group, compared to 2.8% in the Paxlovid group (12). We cannot conclusively establish a correlation between abnormal liver function and VV116 metabolite accumulation in this case, as severe COVID-19 cases can cause transient elevation of transaminases due to increased inflammation (13, 16). It is needed to be vigilant about the potential impact on liver function in cases of severe drug accumulation, additional studies on renal function-referred dosage adjustment are warranted in the future.

## 4 Conclusion

In this case series, it is the first time to demonstrate the therapeutic effects and drug accumulation of VV116 in patients with renal function impairment. Our preliminary findings suggest that using half the dose of VV116 in these patients can maintain effective therapeutic plasma concentrations and significantly reduce drug accumulation. Additionally, no drug-drug interactions were observed between VV116 and tacrolimus. These findings indicate that VV116 has the potential to be a viable antiviral option for COVID-19 in patients who are receiving tacrolimus. Furthermore, it is needed to conduct clinical studies to determine the optimal dosage based on creatinine clearance rate.

## Data Availability

The original contributions presented in the study are included in the article/**Supplementary Material**. Further inquiries can be directed to the corresponding author.
