# Supplementary material for: Severe metabolic accumulation of VV116 in kidney transplant patients with impaired renal function: a case series report
Source: Front Immunol. 2025 Jan 17;15:1501813. doi: 10.3389/fimmu.2024.1501813 (PMC11782228; doi:10.3389/fimmu.2024.1501813)
Supplement: Supplementary file 1 [file Table1.docx]

Supplementary Material

# Bioanalytical procedures for VV116-N1

**Sample Collection**

Blood samples for quantification of VV116-N1 were collected before the first, third, fifth, and seventh administration and at 1 and 2 h after the tenth administration. A 3.5 mL aliquot of blood was drawn into collection systems containing EDTA-K_2_.

**Bioanalysis**

Plasma VV116-N1 concentrations were determined by a validated liquid chromatography/mass spectrometry method. VV116-N1 and its internal standard were provided by Vigonvita Life Science. VV116-N1 and VV116-3-4D were extracted from plasma using 0.3 mL methanol followed by shaking for 10 min and centrifugation at 3500g for 5 min. A 30 μL of supernatant was transferred to a clean tube and diluted with 370 μL of 20% methanol/water. Five microliters of the sample were directly injected into the liquid chromatography/mass spectrometry system. Chromatographic separation was achieved by gradient elution on an Excel 3 C18-PFP 3μm 4.6*50mm (ACE, Aberdeen, Scotland). The mobile phase A consisted of 10 mM NH_4_HCO_3_ in ultra purified water, the mobile phase B was 100% methanol. Compound was separated with Shimadzu LC-20AD HPLC and the detection was accomplished on an API 5000 (Applied Biosystems). The plasma standard curves, which ranged from 0.0100~10.0 μg/mL for VV116-N1, were fitted to a 1/x^2^ weighted regression model. In this case, trough plasma concentration of VV116-N1 after the first, third, fifth, and seventh dose was 2.96, 3.90, 3.62 and 4.41 ug/ml, respectively. Plasma concentration of VV116-N1 at 1 hour and 2 hours after the tenth dose was 4.44 and 4.35 ug/ml, respectively.

# Supplementary Figures and Tables

## Supplementary Figure S1


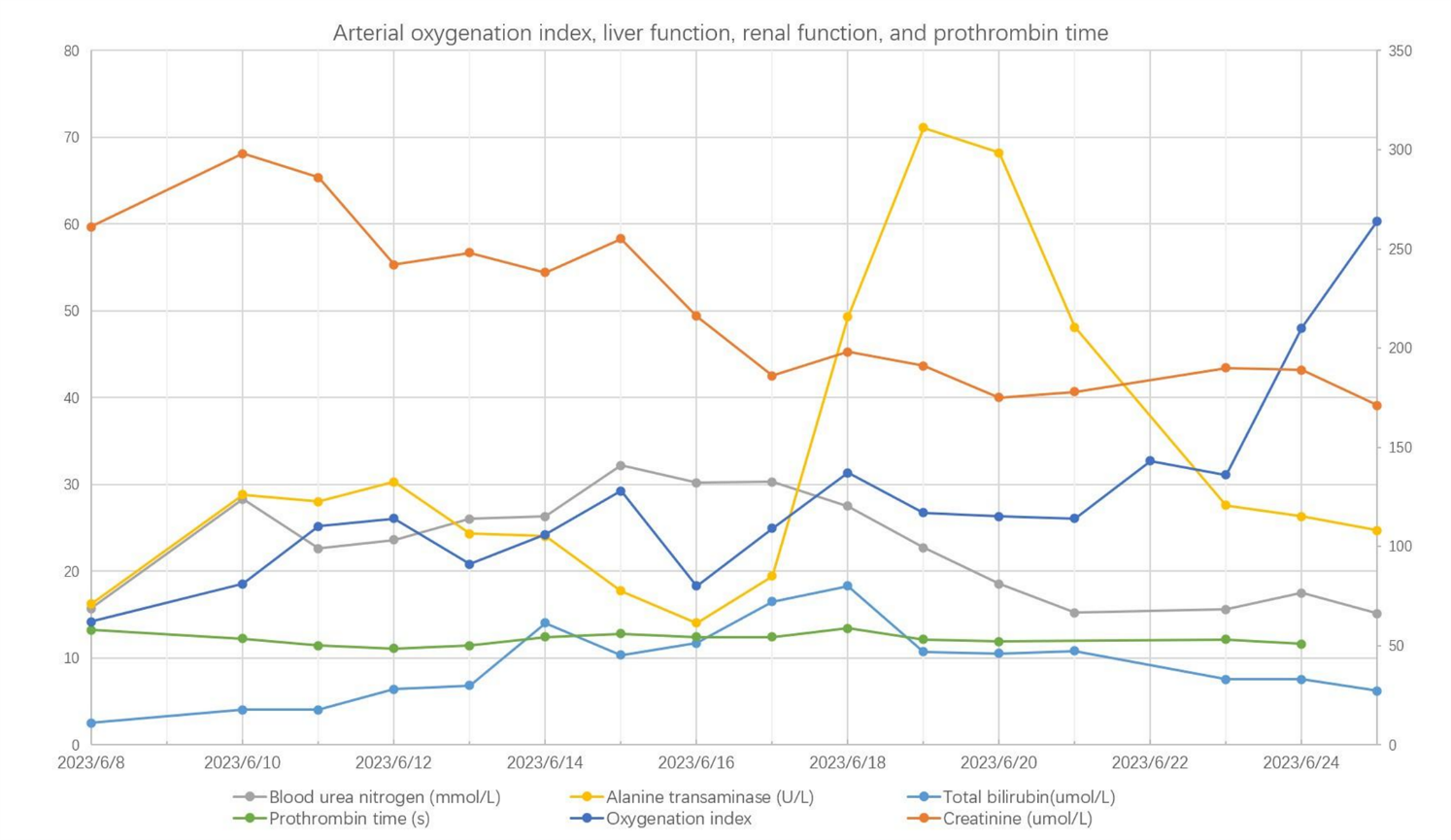


**Figure.S1 Arterial oxygenation index, liver function, renal function, and prothrombin time for Case 1**

Blood urea nitrogen, alanine aminotransferase, total bilirubin, and prothrombin time values correspond to the left vertical axis, while creatinine and arterial oxygenation index values correspond to the right vertical axis.

## Supplementary Table 1. Details of Literature Searching Strategy

| Database | Search strategy |
| --- | --- |
| PubMed | (("renal insufficiency"[All Fields] OR "kidney disease"[All Fields] OR "renal impairment"[All Fields] OR "kidney failure"[All Fields] OR "chronic kidney disease"[All Fields] OR ("transplant patient"[All Fields] OR "organ transplantation"[All Fields] OR "transplant recipient"[All Fields])) AND ("drug interaction"[All Fields] OR "drug-drug interaction"[All Fields]) AND (("sars cov 2"[MeSH Terms] OR "sars cov 2"[All Fields] OR "sars cov 2"[All Fields] OR ("covid 19"[All Fields] OR "covid19"[All Fields] OR "covid 19"[MeSH Terms] OR "covid 19 vaccines"[All Fields] OR "covid 19 vaccines"[MeSH Terms] OR "covid 19 serotherapy"[All Fields] OR "covid 19 serotherapy"[MeSH Terms] OR "covid 19 nucleic acid testing"[All Fields] OR "covid 19 nucleic acid testing"[MeSH Terms] OR "covid 19 serological testing"[All Fields] OR "covid 19 serological testing"[MeSH Terms] OR "covid 19 testing"[All Fields] OR "covid 19 testing"[MeSH Terms] OR "sars cov 2"[All Fields] OR "sarscov2"[All Fields] OR "sarscov 2"[All Fields] OR "sars cov2"[All Fields] OR "sars cov 2"[MeSH Terms] OR "severe acute respiratory syndrome coronavirus 2"[All Fields] OR "2019 ncov"[All Fields] OR (("coronavirus"[MeSH Terms] OR "coronavirus"[All Fields] OR "cov"[All Fields] OR "ncov"[All Fields]) AND 2019/11/01:3000/12/31[Date - Publication]))) AND ("antiviral agents"[Pharmacological Action] OR "antiviral agents"[MeSH Terms] OR ("antiviral"[All Fields] AND "agents"[All Fields]) OR "antiviral agents"[All Fields] OR "antivirals"[All Fields] OR "antiviral"[All Fields] OR "antivirally"[All Fields] OR "antiviral agents"[MeSH Terms] OR "remdesivir"[Title/Abstract] OR "favipiravir"[Title/Abstract] OR "molnupiravir"[Title/Abstract] OR "Paxlovid"[Title/Abstract] OR "nirmatrelvir"[Title/Abstract] OR "VV116"[Title/Abstract] OR "Simnotrelvir"[Title/Abstract]))) NOT "review"[Publication Type] |

## Table2.The Summary of application of COVID-19 antiviral agents in patients with solid organ transplantation (The retrieval strategy for this table is placed in the appendix)

| Author （year） | Study type | No. of  participants | Intervention | Result |
| --- | --- | --- | --- | --- |
| Kristin Stawiarski *et al*. (2023)(Stawiarski et al., 2023) | Case Reports | 1 | nirmatrelvir/ritonavir | Tacrolimus level returned supratherapeutic at 49 ng/mL from 5.5 ng/mL. The patient also had acute kidney injury with creatinine of 2.6 mg/dL, from baseline 1.8-2.0 mg/dL. |
| Ri Ra *et al*. (2021)(Ra et al., 2021) | Case Reports | 1 | lopinavir/ritonavir | Sirolimus trough level abruptly increased to 122.9 ng/mL and induced hepatitis. |
| Yaerim Kim *et al.*(2020)(Kim et al., 2020) | Case Reports | 2 | lopinavir/ritonavir | Researcher used lopinavir/ritonavir and hydroxychloroquine for the first case and hydroxychloroquine for the second case. For the first case, the tacrolimus trough level peaked at 24.6 ng/mL . |
| Toshinori Hirai *et al*.(Hirai et al., 2022) | Case Reports | 1 | remdesivir | After starting remdesivir on day 4, the increased trough concentrations of tacrolimus on day 6 (6.9 ng/mL) and everolimus on day 7 (9.2 ng/mL) were observed. |
| Soufian Meziyerh *et al*. (2020)(Meziyerh et al., 2020) | Case Reports | 1 | [chloroquine](https://www.sciencedirect.com/topics/pharmacology-toxicology-and-pharmaceutical-science/chloroquine) and lopinavir/ritonavir | The clinical course was complicated by extreme overexposure to the everolimus. |
| Wei Luo *et al*. (2023)(Luo et al., 2023) | Case Reports | 1 | nirmatrelvir/ritonavir | Caused severe diarrhea, combined diabetic ketoacidosis and a hyperglycemic hyperosmolar state |
| David M Salerno *et al*. (2022) (Salerno et al., 2022) | Retrospective study | 25 | nirmatrelvir/ritonavir | Four patients experienced a supratherapeutic tacrolimus concentration. |
| Marios Prikis *et al*. (2022)(Prikis and Cameron, 2022) | Case Reports | 1 | nirmatrelvir/ritonavir | Leading to an abruptly high tacrolimus level, significant nausea and vomiting, treatment interruption, and acute kidney injury. |
| Noa Berar Yanay *et al*. (2022)(Berar Yanay et al., 2022) | Case Reports | 1 | nirmatrelvir/ritonavir | The tacrolimus trough blood concentration increased up to 92.4 ng/mL on the fourth day after the first nirmatrelvir/ritonavir dose (2 days after nirmatrelvir/ritonavir was stopped). |
| Ruben Gonzalez-García *et al.* (2023).(Gonzalez-García et al., 2023) | Observational Study | 23 | lopinavir/ritonavir | Nine of the 21 (42.8%) patients on tacrolimus treatment had C_0_ above the cutoff point after lopinavir/ritonavir initiation. Three patients had very high concentrations (>40 ng/mL) and developed toxicity. |
| Takeshi Tomida *et al*. (2023)(Tomida et al., 2023) | Experimental study | 1 | nirmatrelvir/ritonavir | A renal transplant patient taking oral tacrolimus continuously was treated with nirmatrelvir/ritonavir for 5 days. The tacrolimus trough concentration increased to 96.4 ng/mL on Day 7. |
| Marilù Bartiromo *et al*. (2020)(Bartiromo et al., 2020) | Case Reports | 1 | Hydroxychloroquine and lopinavir/ritonavir were started, replaced with darunavir/cobicistat after 2 days for diarrhea. | Researchers observed very high tacrolimus trough levels despite initial dose reduction. |
| Ayumi Tsuzawa *et al*. (2023)(Tsuzawa et al., 2023) | Case Reports | 1 | nirmatrelvir/ritonavir | Tacrolimus was resumed at 1.0 mg/d after completion of the nirmatrelvir/ritonavir treatment, but the trough value after 7 d was high at 31.6 ng/ml. |
| Chelsea Young *et al*. (2023)(Young et al., 2023) | Case Reports | 1 | nirmatrelvir/ritonavir | The patient subsequently develops supratherapeutic tacrolimus levels and an increase in serum creatinine. |
| Sujal Modi *et al*. (2023)(Modi et al., 2023) | Case Reports | 1 | nirmatrelvir/ritonavir | Serum chemistry noted a mildly elevated creatinine level of 1.31 mg/dL with a blood urea nitrogen of 31 mg/dL. Serum tacrolimus level was recorded as >60 ng/mL. |
| Thanawat Vongchaiudomchoke *et al*. (2021) (Vongchaiudomchoke et al., 2021) | Case Reports | 1 | favipiravir and lopinavir/ritonavir | Tacrolimus trough concentration taken 7 days after starting lopinavir/ritonavir which returned 7 days later was 66.3 ng/mL. The patient's serum creatinine peaked at 7.0 mg/dL. |
| Theerachai Thammathiwat *et al*. (2020)(Thammathiwat et al., 2021) | Case Reports | 1 | favipiravir | The laboratory results showed rising of Cr from 1.4 and the trough level of tacrolimus was 49.4 ng/mL |
| Yu Xiong *et al*. (2023)(Xiong et al., 2023) | Case Reports | 1 | nirmatrelvir/ritonavir | The patient presented to the hospital with symptoms of headache, dizziness, palpitations, abdominal distension, nausea, vomiting, and diarrhea，and the blood concentration of tacrolimus was measured at 106 ng/ml. |
| Chien-Ming Lo *et al*. (2024)(Lo et al., 2024) | Case Reports | 1 | nirmatrelvir/ritonavir | The patient experienced hyponatremia and showed a high serum tacrolimus concentration. |
| Hikari Yoshida *et al*. (2024)(Yoshida et al., 2024) | Case Reports | 1 | nirmatrelvir/ritonavir | The patient developed diarrhea and vomiting and was hospitalized. Tacrolimus was discontinued on day 6, and tacrolimus level was measured on day 8 and had risen above 100 ng/mL. |

Bartiromo, M., Borchi, B., Botta, A., Bagalà, A., Lugli, G., Tilli, M., et al. (2020). Threatening drug-drug interaction in a kidney transplant patient with coronavirus disease 2019 (COVID-19). *Transplant Infectious Disease : an Official Journal of the Transplantation Society* 22(4)**,** e13286. doi: 10.1111/tid.13286.

Berar Yanay, N., Bogner, I., Saker, K., and Tannous, E. (2022). Paxlovid-Tacrolimus Drug-Drug Interaction in a 23-Year-Old Female Kidney Transplant Patient with COVID-19. *Clinical Drug Investigation* 42(8)**,** 693-695. doi: 10.1007/s40261-022-01180-4.

Gonzalez-García, R., Roma, J.-R., Rodríguez-García, M., Arranz, N., Ambrosioni, J., Bodro, M., et al. (2023). Drug-drug interactions of ritonavir-boosted SARS-CoV-2 protease inhibitors in solid organ transplant recipients: experience from the initial use of lopinavir-ritonavir. *Clinical Microbiology and Infection : the Official Publication of the European Society of Clinical Microbiology and Infectious Diseases* 29(5)**,** 655.e651-655.e654. doi: 10.1016/j.cmi.2023.01.002.

Hirai, T., Mizuta, A., Sasaki, T., Nishikawa, K., Inoue, T., and Iwamoto, T. (2022). Drug-drug interaction between remdesivir and immunosuppressant agents in a kidney transplant recipient. *International Journal of Clinical Pharmacology and Therapeutics* 60(10)**,** 439-444. doi: 10.5414/CP204239.

Kim, Y., Kwon, O., Paek, J.H., Park, W.Y., Jin, K., Hyun, M., et al. (2020). Two distinct cases with COVID-19 in kidney transplant recipients. *American Journal of Transplantation : Official Journal of the American Society of Transplantation and the American Society of Transplant Surgeons* 20(8)**,** 2269-2275. doi: 10.1111/ajt.15947.

Lo, C.-M., Chen, W.-H., Tsai, M.-Y., Lu, H.-I., Hsiao, Y.-H., Chuang, K.-H., et al. (2024). A case report of drug interaction between co-packaged nirmatrelvir-ritonavir and tacrolimus causing hyponatremia in a lung transplant recipient. *Journal of Cardiothoracic Surgery* 19(1)**,** 132. doi: 10.1186/s13019-024-02599-w.

Luo, W., He, Y., Wei, M.G., Lu, G.B., and Yi, Q. (2023). Paxlovid-tacrolimus drug-drug interaction caused severe diarrhea that induced combined diabetic ketoacidosis and a hyperglycemic hyperosmolar state in a kidney transplant patient: a case report. *Journal of Medical Case Reports* 17(1)**,** 406. doi: 10.1186/s13256-023-04135-1.

Meziyerh, S., Zwart, T.C., van Etten, R.W., Janson, J.A., van Gelder, T., Alwayn, I.P.J., et al. (2020). Severe COVID-19 in a renal transplant recipient: A focus on pharmacokinetics. *American Journal of Transplantation : Official Journal of the American Society of Transplantation and the American Society of Transplant Surgeons* 20(7)**,** 1896-1901. doi: 10.1111/ajt.15943.

Modi, S., Kahwash, R., and Kissling, K. (2023). Case Report: tacrolimus toxicity in the setting of concurrent Paxlovid use in a heart-transplant recipient. *European Heart Journal. Case Reports* 7(5)**,** ytad193. doi: 10.1093/ehjcr/ytad193.

Prikis, M., and Cameron, A. (2022). Paxlovid (Nirmatelvir/Ritonavir) and Tacrolimus Drug-Drug Interaction in a Kidney Transplant Patient with SARS-2-CoV infection: A Case Report. *Transplantation Proceedings* 54(6)**,** 1557-1560. doi: 10.1016/j.transproceed.2022.04.015.

Ra, R., Kim, J.S., Jeong, K.H., and Hwang, H.S. (2021). COVID-19 and Sirolimus Treatment in a Kidney Transplant Recipient. *Experimental and Clinical Transplantation : Official Journal of the Middle East Society For Organ Transplantation* 19(9)**,** 977-980. doi: 10.6002/ect.2021.0232.

Salerno, D.M., Jennings, D.L., Lange, N.W., Kovac, D.B., Shertel, T., Chen, J.K., et al. (2022). Early clinical experience with nirmatrelvir/ritonavir for the treatment of COVID-19 in solid organ transplant recipients. *American Journal of Transplantation : Official Journal of the American Society of Transplantation and the American Society of Transplant Surgeons* 22(8)**,** 2083-2088. doi: 10.1111/ajt.17027.

Stawiarski, K., Avery, R., Strout, S., and Umapathi, P. (2023). Risks of paxlovid in a heart transplant recipient. *J Heart Lung Transplant* 42(1)**,** 30-32. doi: 10.1016/j.healun.2022.08.029.

Thammathiwat, T., Tungsanga, S., Tiankanon, K., Torvorapanit, P., Chumpangern, W., Udomkarnjananun, S., et al. (2021). A case of successful treatment of severe COVID-19 pneumonia with favipiravir and tocilizumab in post-kidney transplant recipient. *Transplant Infectious Disease : an Official Journal of the Transplantation Society* 23(1)**,** e13388. doi: 10.1111/tid.13388.

Tomida, T., Itohara, K., Yamamoto, K., Kimura, T., Fujita, K., Uda, A., et al. (2023). A model-based pharmacokinetic assessment of drug-drug interaction between tacrolimus and nirmatrelvir/ritonavir in a kidney transplant patient with COVID-19. *Drug Metabolism and Pharmacokinetics* 53**,** 100529. doi: 10.1016/j.dmpk.2023.100529.

Tsuzawa, A., Katada, Y., Umemura, K., Sugimoto, M., Nishikawa, A., Sato, Y.-K., et al. (2023). A case report of a prolonged decrease in tacrolimus clearance due to co-administration of nirmatrelvir/ritonavir in a lung transplant recipient receiving itraconazole prophylaxis. *Journal of Pharmaceutical Health Care and Sciences* 9(1)**,** 12. doi: 10.1186/s40780-023-00280-3.

Vongchaiudomchoke, T., Sawangduan, V., Sinpanee, T., Chalermphunchai, N., Lelamali, K., and Noppakun, K. (2021). Modification of immunosuppressive agents in a kidney transplant recipient with COVID-19 and acute kidney injury. *Journal of Infection In Developing Countries* 15(9)**,** 1273-1276. doi: 10.3855/jidc.13176.

Xiong, Y., Wang, X., Li, S., Zhang, Q., Guo, L., Chen, W., et al. (2023). Case report: Supratherapeutic tacrolimus concentrations with nirmatrelvir/ritonavir in a lung transplant patient: a case report using Rifampin for reversal. *Frontiers In Pharmacology* 14**,** 1285078. doi: 10.3389/fphar.2023.1285078.

Yoshida, H., Umemura, T., Ito, S., Mizuno, T., Mutoh, Y., Yamada, T., et al. (2024). Abnormally Elevated Blood Tacrolimus Level Following the Concomitant Use of Nirmatrelvir/Ritonavir With Extended-Release Tacrolimus in a Post-lung Transplant Patient: A Case Report and a Literature Review. *Cureus* 16(6)**,** e62868. doi: 10.7759/cureus.62868.

Young, C., Papiro, T., and Greenberg, J.H. (2023). Elevated tacrolimus levels after treatment with nirmatrelvir/ritonavir (Paxlovid) for COVID-19 infection in a child with a kidney transplant. *Pediatric Nephrology (Berlin, Germany)* 38(4)**,** 1387-1388. doi: 10.1007/s00467-022-05712-0.

**
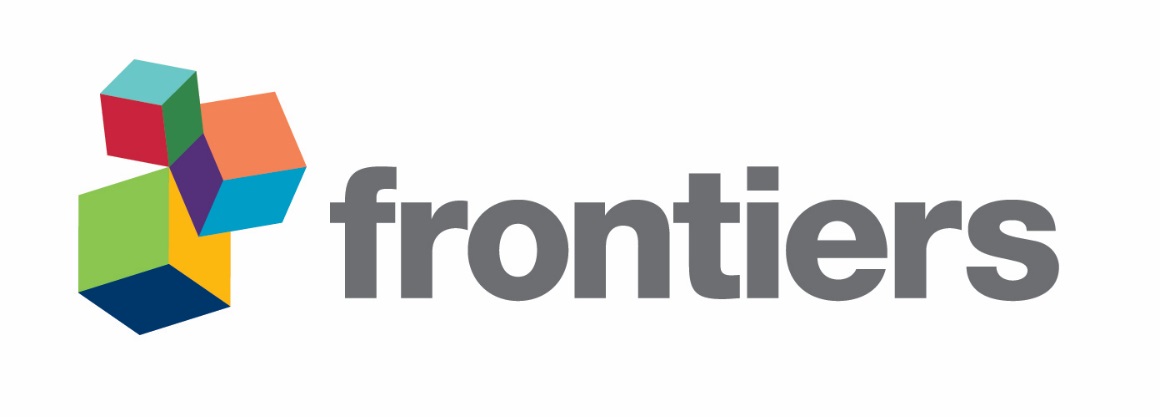
**
